# The Weibull-Gamma Distribution: Properties and Applications

**DOI:** 10.3390/e21050438

**Published:** 2019-04-26

**Authors:** Hadeel S. Klakattawi

**Affiliations:** Department of Statistics, Faculty of Science, King Abdulaziz University, Jeddah 21589, Saudi Arabia; hklakattawi@kau.edu.sa

**Keywords:** weibull distribution, gamma distribution, transformed-transformer family of distributions, maximum likelihood estimation, simulation study

## Abstract

A new member of the Weibull-generated (Weibull-G) family of distributions—namely the Weibull-gamma distribution—is proposed. This four-parameter distribution can provide great flexibility in modeling different data distribution shapes. Some special cases of the Weibull-gamma distribution are considered. Several properties of the new distribution are studied. The maximum likelihood method is applied to obtain an estimation of the parameters of the Weibull-gamma distribution. The usefulness of the proposed distribution is examined by means of five applications to real datasets.

## 1. Introduction

Probability distributions provide important information about the statistical inference and analysis of data. These results can be used for considering some well-grounded decisions. Hence, it is essential to have a distribution that accurately reflects the data. Different classical distributions have been broadly applied for the description of real-world phenomena and the modeling of data in different disciplines, including insurance, economics, finance, engineering, biology, industry, and medical sciences. However, many of these standard distributions have limitations in fitting some of the real data accurately. Therefore, it is of great importance to identify which distribution should be applied to model the data. Knowledge of the appropriate distribution greatly improves the efficiency of any statistical inference related to data sets. Hence, researchers are always trying to extend existing classical distributions, to improve their goodness-of-fit and obtain more flexibility and adaptability in modeling data in practice. This paper proposes a new modified distribution, which has increased flexibility to fit various data in practice. Many approaches to extend the existing standard models and develop generalized classes of distributions have been suggested in the literature; for an excellent review of various approaches in generating distributions, one may refer to [1].

Recently, Alzaatreh et al. [2] proposed a new approach for generating families of distributions, namely the transformed-transformer (T-X) family of distributions. These families can be defined for any baseline distribution with probability density function (pdf) *g*, cumulative density function (cdf) *G*, and a parameter vector ζ, by applying a function to its cdf. To illustrate, assume a continuous generator random variable (RV) *T* is defined on [a,b] with pdf r(t) and cdf R(t). Then, the cdf of the T-X family of distributions for a RV *X* is defined as

$$F\left(x\right)=\int_{a}^{W(G(x;\zeta ))}r\left(t\right)dt,$$

which can be written as

(1) F(x)=R(W(G(x;ζ))),

where W(G(x;ζ) is a function of the cdf *G* which satisfies W(G(x;ζ)∈[a,b], W(G(x;ζ) is differentiable and monotonically non-decreasing, W(G(x;ζ)→a as x→−∞, and W(G(x;ζ)→b as x→∞. The corresponding pdf, associated with Equation (1), can be found as

$$f\left(x\right)=\left\{\frac{d}{dx}W\left(G\left(x;\zeta \right)\right)\right\}r\left(W\left(G\left(x;\zeta \right)\right)\right).$$

Different forms of the upper limit *W* can be used to generate different types of the T-X family of distributions. Additionally, the term “generated” (which we will denote G, for short) illustrates that for each baseline distribution *G*, a different distribution *F* can be obtained; that is, for each family, several sub-models can be derived according to the choice of the distribution *G*. For more details, see [2], where they choose W(G(xζ))=−log(1−(G(xζ))) in order to introduce the gamma-G, beta-exponential-G, and Weibull-G families. Many T-X generated distributions have been proposed recently. For example, the gamma-G family was introduced in [3], the Lomax-G family was introduced in [4], the Lindley-G family was introduced in [5], the Gompertz-G family was introduced in [6], the generalized Burr-G family was introduced in [7], the power Lindley-G family was introduced in [8], and the odd Lomax-G family was introduced in [9], among others.

The gamma distribution can be considered to be one of the most commonly applied lifetime distributions in different fields. A RV *X* is said to have a gamma distribution, with shape parameter k>0 and a scale parameter s>0, if the pdf and cdf of *X* are, respectively, given as

(2) $$g\left(x;k,s\right)=\frac{1}{s^{k}\Gamma \left(k\right)}x^{k-1}e^{-\frac{x}{s}}\;;\;x\geq 0$$

and

(3) $$G\left(x;k,s\right)=\frac{\gamma (k,\frac{x}{s})}{\Gamma \left(k\right)},$$

where Γ(k) is the gamma function and γ(k,x) is the incomplete gamma function. given respectively as

(4) $$\Gamma \left(k\right)=\int_{0}^{\infty }t^{k-1}e^{-t}dt\;and\;\gamma \left(k,\frac{x}{s}\right)=\int_{0}^{\frac{x}{s}}t^{k-1}e^{-t}dt.$$

Many attempts have been made to increase the flexibility of this distribution by introducing some new distribution with additional parameter(s), such as the exponentiated gamma (EG). This distribution can be considered to be a member of the exponentiated class of distributions proposed in [10], where the cdf of any classical distribution is raised to a power (shape) parameter. Thus, the cdf of the EG takes the form

(5) $$F\left(x;s,k,\alpha \right)={\left[\frac{\gamma (k,\frac{x}{s})}{\Gamma \left(k\right)}\right]}^{\alpha }.$$

See, for example [11], where the one-parameter gamma (with scale parameter s=1), is exponentiated.

This paper proposes a new modification of the gamma distribution, based on the Weibull-G (W-G) family of distributions. The additional parameters presented by the Weibull generator might enhance the flexibility of the distribution. The rest of the paper is organized as follows. In Section 2, the W-G family is discussed. A member of this family—namely the Weibull-gamma (W-g) distribution—is introduced in Section 3. Some special cases of the W-g distribution are examined in Section 4. In Section 5, some of the properties of the new distribution are briefly discussed. The method of maximum likelihood for estimating the parameters of the W-g distribution is discussed in Section 6. Then, the consistency and precision of these estimates, by means of some Monte Carlo simulation studies, are investigated in Section 7. Finally, five real datasets are applied, in Section 8, to investigate the flexibility and usefulness of the W-g distribution.

## 2. The Weibull-Generated Family

A RV *T* is said to have a Weibull distribution, with shape parameter c>0 and scale parameter β>0, if its pdf and cdf are, respectively, given by

(6) $$r\left(t;c,\beta \right)=\frac{c}{\beta }{\left(\frac{t}{\beta }\right)}^{c-1}e^{-{\left(\frac{t}{\beta }\right)}^{c}}\;;\;t\geq 0,\;\mathrm{and}$$

(7) $$R\left(t;c,\beta \right)=1-e^{-{\left(\frac{t}{\beta }\right)}^{c}}.$$

Assuming that G(x;ζ) is a cdf of the baseline distribution with parameter vector ζ, the cdf of the W-G distribution can be derived by replacing *t* in Equation (7) by W(G(x;ζ)), as follows

(8) $$\begin{array}{cc} F(x,c,\beta ,\zeta ) & =\int_{0}^{W(G(x;\zeta ))}\frac{c}{\beta }{\left(\frac{t}{\beta }\right)}^{c-1}e^{-{\left(\frac{t}{\beta }\right)}^{c}}dt\\ & =1-e^{-{\left(\frac{W(G(x;\zeta ))}{\beta }\right)}^{c}}. \end{array}$$

Then, the W-G distribution is obtained with two extra parameters, *c* and β, for any baseline distribution *G* distribution. Different types of W-G can be found, based on the choice of the upper limit of the integral W(G(x;ζ)). In other words, ref [12] assumed $W\left(G\left(x\right)\right)=-log\left(1-G^{\alpha }\left(x;\zeta \right)\right)$ for α>0 to introduce the exponentiated W-G, ref [13] considered $W\left(G\left(x;\zeta \right)\right)=\frac{G(x;\zeta )}{1-G(x;\zeta )}$, ref [14] used the form of $W\left(G\left(x;\zeta \right)\right)=\frac{1}{1-G(x;\zeta )}$, and [15] assumed W(G(x;ζ))=−log(G(x;ζ)). In this paper, W(G(x;ζ))=−log(1−G(x;ζ)), which was discussed by [2,16,17], is considered in particular. The cdf of the W-G distribution is defined as

(9) $$\begin{array}{cc} F(x;c,\beta ,\zeta ) & =\int_{0}^{-log(1-G(x;\zeta ))}\frac{c}{\beta }{\left(\frac{t}{\beta }\right)}^{c-1}e^{-{\left(\frac{t}{\beta }\right)}^{c}}dt\\ & =1-e^{-{\left(\frac{-log(1-G(x;\zeta ))}{\beta }\right)}^{c}}, \end{array}$$

with the corresponding pdf

(10) $$f\left(x;c,\beta ,\zeta \right)=\frac{c}{\beta }\left(\frac{g(x;\zeta )}{1-G(x;\zeta )}\right){\left(\frac{-log(1-G(x;\zeta ))}{\beta }\right)}^{c-1}e^{-{\left(\frac{-log(1-G(x;\zeta ))}{\beta }\right)}^{c}}.$$

In [18], a transformer *X* distributed as Pareto distribution was considered, to introduce the Weibull-Pareto distribution. In [16], the logistic distribution was used as a baseline distribution, providing the Weibull-logistic model. The Weibull-log-logistic distribution was discussed in [17] as a special case of the W-G family of distributions. Additionally, ref [19] applied this form of the upper limit for the Rayleigh and discussed the Weibull-Rayleigh distribution.

## 3. The Weibull-Gamma Distribution

The W-g distribution is derived as a member of the W-G family of distributions in Equation (9); that is, *T* is a Weibull RV and *X* is a gamma RV. Then, the pdf and cdf of the W-g distribution, with a vector of parameters $\zeta =\left\{c,\beta ,k,s\right\}$, can be found by substituting with Equations (2) and (3) in Equations (9) and (10), as follows

(11) $$\begin{array}{cc} f(x;c,\beta ,k,s)= & \frac{c}{\beta s^{k}\Gamma \left(k\right)}\frac{x^{k-1}e^{-\frac{x}{s}}}{w(x;k)}{\left(\frac{-log(w(x;k))}{\beta }\right)}^{c-1}\times \\ & exp\left(-{\left(\frac{-log(w(x;k))}{\beta }\right)}^{c}\right) \end{array}$$

and

(12) $$F\left(x;c,\beta ,k,s\right)=1-exp\left(-{\left(\frac{-log(w(x;k))}{\beta }\right)}^{c}\right).$$

The reliability function of the W-g can be obtained, consequently, as

(13) $$R\left(x;c,\beta ,k,s\right)=1-F\left(x;c,\beta ,k,s\right)=exp\left(-{\left(\frac{-log(w(x;k))}{\beta }\right)}^{c}\right),$$

where x≥0, c,β,k,s>0, and

(14) $$w\left(x;k\right)=1-\frac{\gamma (k,\frac{x}{s})}{\Gamma (k)}.$$

The hazard function can be defined as

(15) $$h\left(x;c,\beta ,k,s\right)=\frac{f(x;c,\beta ,k,s)}{1-F(x;c,\beta ,k,s)},$$

where f(x) and F(x) are, respectively, defined by Equations (11) and (12).

**Figure 1:**
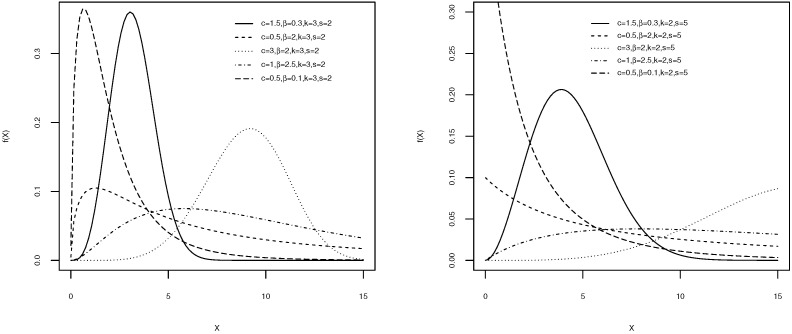

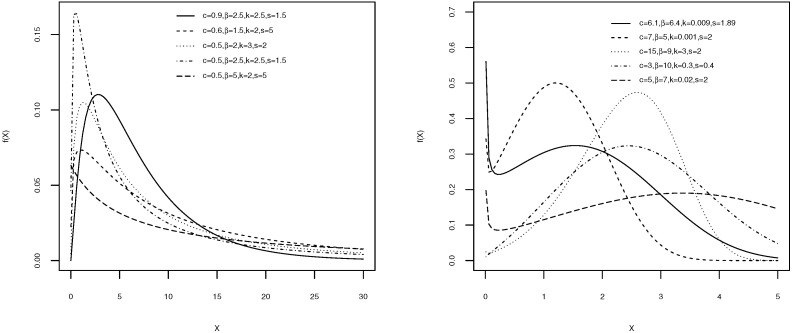
The Weibull-gamma (W-g) probability density functions (pdfs) for various values of *c*, β, *k*, and *s*.

**Figure 2:**
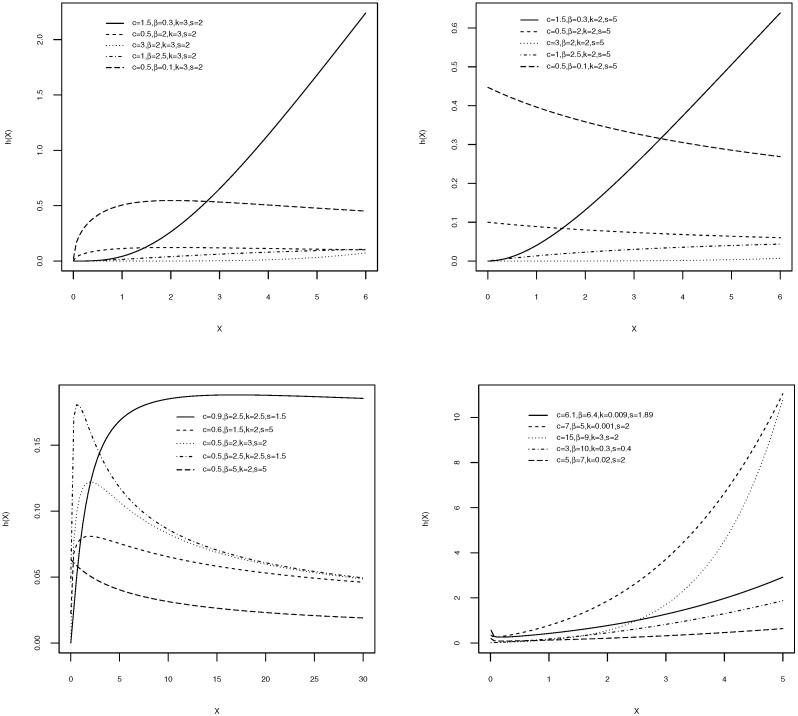
The W-g hazard functions for various values of *c*, β, *k*, and *s*.

Different plots of the pdf and the hazard functions for the W-g distribution are displayed, respectively, in Figure 1 and Figure 2, for some specific parameter values. The density and hazard functions show differing behaviors, based on the values of the parameters. The various possible shapes of the density function, including (approximately) symmetric, skewed, and bimodal, were produced. Additionally, several shapes, including monotonically decreasing, monotonically increasing, unimodal, bathtub, and U shapes, can be obtained for the hazard function of the W-g, for different combinations of the values of the parameters. This illustrates the great flexibility of the W-g distribution, which make it suitable for various real data.

## 4. Some Special Cases of the Weibull-Gamma Distribution

- If c=β=k=s=1, the W-g distribution reduces to the standard exponential distribution, with pdf as follows
  $$f\left(x\right)=e^{-x}\;;\;x>0.$$
- When c=β=1 in the W-g model, the gamma distribution in Equation (2) with shape parameter *k* and scale parameter *s* is obtained.
- If c=1, the W-g distribution reduces to the exponential-gamma distribution, with pdf as follows
  $$f\left(x;\beta ,k,s\right)=\frac{x^{k-1}e^{-\frac{x}{s}}}{\beta s^{k}\Gamma \left(k\right)}{\left(1-\frac{\gamma (k,\frac{x}{s})}{\Gamma (k)}\right)}^{\frac{1}{\beta }-1}\;;\;x>0,$$
  where Γ(k) and γ(k,x) are, respectively, defined by Equation (4).

## 5. Properties

Providing some mathematical expansions to find some characteristics of the W-g distribution might be more reasonable than numerically solving the integrals of the pdf given by Equation (11) to derive these properties. Hence, some mathematical properties are provided here using algebraic expansions, which can be carried out using any computational software platform which can deal with analytic expressions.

### 5.1. Useful Expansions

In the following, we show an alternative formula for the pdf of the W-g distribution given in Equation (11). Using the power series for the exponential function

$$e^{-x}=\sum_{a_{1}=0}^{\infty }\frac{{\left(-1\right)}^{a_{1}}}{a_{1}!}x^{a_{1}},$$

we obtain

$$f\left(x;c,\beta ,k,s\right)=\frac{c}{\beta s^{k}\Gamma \left(k\right)}\frac{x^{k-1}e^{-\frac{x}{s}}}{w(x;k)}\sum_{a_{1}=0}^{\infty }\frac{{\left(-1\right)}^{a_{1}}}{a_{1}!}{\left(\frac{-log(w(x;k))}{\beta }\right)}^{a_{1}c+c-1}.$$

Applying the binomial theorem, which defines ${\left(1-x\right)}^{-1}$ as

$${\left(1-x\right)}^{-1}=\sum_{a_{2}=0}^{\infty }x^{a_{2}},$$

to expand $w{\left(x;k\right)}^{-1}$ (where the definition of w(x;k) is given in Equation (14)), the pdf can be reduced to

$$\begin{array}{cc} f\left(x;c,\beta ,k,s\right)=\frac{c}{\beta s^{k}\Gamma \left(k\right)} & x^{k-1}e^{-\frac{x}{s}}\sum_{a_{2}=0}^{\infty }{\left(\frac{\gamma (k,\frac{x}{s})}{\Gamma (k)}\right)}^{a_{2}}\times \\ & \sum_{a_{1}=0}^{\infty }\frac{{\left(-1\right)}^{a_{1}}}{a_{1}!}{\left(\frac{1}{\beta }\right)}^{a_{1}c+c-1}{\left(-log\left(1-\frac{\gamma (k,\frac{x}{s})}{\Gamma (k)}\right)\right)}^{a_{1}c+c-1}. \end{array}$$

Furthermore, ref [17,20] applied the generalized binomial theorem to prove that

−log(1−x)a=a∑a3=0∞∑a4=0a3(−1)a4+a3a3−aa3a3a4pa4,a3(a−a4)xa+a3.

Consequently, the pdf can be obtained as

f(x;c,β,k,s)=cβskΓ(k)xk−1e−xs×∑a1,a2,a3=0∞∑a4=0a3(−1)a1+a3+a4(a1c+c−1)a3−a1c−c+1a3a3a4pa4,a3(a1!)βa1c+c−1(a1c+c−a4−1)×γ(k,xs)Γ(k)a1c+c+a2+a3−1,

where the constant $p_{a_{4},a_{3}}$ can be found recursively by

$$p_{a_{4},a_{3}}=a_{3}^{-1}\sum_{l=1}^{a_{3}}\left(a_{3}-l\left(a_{4}+1\right)\right)c_{l}p_{a_{4},a_{3}-l},$$

for $a_{3}=1,2,\ldots$, $p_{a_{4},0}=1$, and $c_{a_{3}}={\left(-1\right)}^{a_{3}+1}{\left(a_{3}+1\right)}^{-1}$.

By application of the expansion for the incomplete gamma function γ(a,x) using the power series, presented in [21] as

$$\gamma \left(a,x\right)=x^{a}\sum_{a_{5}=0}^{\infty }\frac{{\left(-1\right)}^{a_{5}}x^{a_{5}}}{a_{5}!\left(a+a_{5}\right)},$$

we obtain

f(x;c,β,k,s)=cβskΓ(k)xk−1e−xs×∑a1,a2,a3=0∞∑a4=0a3(−1)a1+a3+a4(a1c+c−1)a3−a1c−c+1a3a3a4pa4,a3(a1!)βa1c+c−1(a1c+c−a4−1)xsk(a1c+c+a2+a3−1)×1Γ(k)a1c+c+a2+a3−1∑a5=0∞(−1)a5xsa5a5!(k+a5)a1c+c+a2+a3−1.

Again, according to [21], the power series raised to an integer *m* can be simplified as

$${\left(\sum_{a_{5}=0}^{\infty }a_{a_{5}}x^{a_{5}}\right)}^{m}=\sum_{a_{5}=0}^{\infty }q_{a_{5}}x^{a_{5}},$$

where $q_{0}={a_{0}}^{m}$ and $q_{v}=\frac{1}{va_{0}}\sum_{a_{5}=1}^{v}\left(a_{5}m-v+a_{5}\right)a_{a_{5}}q_{v-a_{5}}$ for v≥1.

Hence, assuming $a_{a_{5}}=\frac{{\left(-1\right)}^{a_{5}}}{a_{5}!\left(k+a_{5}\right)}$ yields

f(x;c,β,k,s)=ce−xs×∑a1,a2,a3,a5=0∞∑a4=0a3(−1)a1+a3+a4(a1c+c−1)a3−a1c−c+1a3a3a4pa4,a3qa5xk(a1c+c+a2+a3)+a5−1)(Γ(k))a1c+c+a2+a3(a1!)sk(a1c+c+a2+a3)+a5βa1c+c(a1c+c−a4−1),

where $q_{0}={a_{0}}^{a_{1}c+c+a_{2}+a_{3}-1}$ and $q_{v}=\frac{1}{va_{0}}\sum_{a_{5}=1}^{v}\left(a_{5}\left(a_{1}c+c+a_{2}+a_{3}\right)-v\right)a_{a_{5}}q_{v-a_{5}}$ for v≥1.

If we define

(16) Aa1,a2,a3,a4,a5=c(−1)a1+a3+a4(a1c+c−1)a3−a1c−c+1a3a3a4pa4,a3qa5(Γ(k))a1c+c+a2+a3(a1!)sk(a1c+c+a2+a3)+a5βa1c+c(a1c+c−a4−1),

and using *A* instead of $A_{a_{1},a_{2},a_{3},a_{4},a_{5}}$ for short, the pdf of the W-g can be rewritten as

(17) $$f\left(x;c,\beta ,k,s\right)=\sum_{a_{1},a_{2},a_{3},a_{5}=0}^{\infty }\sum_{a_{4}=0}^{a_{3}}Ax^{k\left(a_{1}c+c+a_{2}+a_{3}\right)+a_{5}-1)}e^{-\frac{x}{s}}.$$

Using a similar technique, the cdf of the W-g distribution can be obtained as

(18) F(x;c,β,k,s)=1−∑a1,a2,a3,a5=0∞∑a4=0a3(−1)a1+a3+a4(a1c)a3−a1ca3a3a4pa4,a3qa5(Γ(k))a1c+a3(a1!)βa1c(a1c+c−a4−1)xsk(a1c+a3)+a5).

### 5.2. Quantile Function

The *p*th quantile function (0<p<1) of the RV *X* which follows the W-g distribution is obtained by inverting Equation (12) and solving the non-linear equation

(19) $$\gamma \left(k,\frac{x}{s}\right)=\Gamma \left(k\right)\left(1-e^{-\beta {\left(-log(1-p)\right)}^{\frac{1}{c}}}\right).$$

### 5.3. Moments

From Equation (17), the *r*th moment of a RV *X* which follows the W-g distribution can be obtained as

(20) $$\begin{array}{cc} \mu_{r}=E\left(X^{r}\right)= & \sum_{a_{1},a_{2},a_{3},a_{5}=0}^{\infty }\sum_{a_{4}=0}^{a_{3}}A\int_{0}^{\infty }x^{r+k\left(a_{1}c+c+a_{2}+a_{3}\right)+a_{5}-1)}e^{-\frac{x}{s}}dx\\ =\sum_{a_{1},a_{2},a_{3},a_{5}=0}^{\infty } & \sum_{a_{4}=0}^{a_{3}}As^{r+k\left(a_{1}c+c+a_{2}+a_{3}\right)+a_{5}}\Gamma \left(r+k\left(a_{1}c+c+a_{2}+a_{3}\right)+a_{5}\right). \end{array}$$

### 5.4. Moment Generating Function

The moment generating function for the W-g distribution follows, from Equation (17), as

(21) $$\begin{array}{cc} M_{x}\left(t\right)= & E\left(e^{tX}\right)=\sum_{a_{1},a_{2},a_{3},a_{5}=0}^{\infty }\sum_{a_{4}=0}^{a_{3}}A\int_{0}^{\infty }x^{k\left(a_{1}c+c+a_{2}+a_{3}\right)+a_{5}-1)}e^{-x(\frac{1}{s}-t)}dx\\ & =\sum_{a_{1},a_{2},a_{3},a_{5}=0}^{\infty }\sum_{a_{4}=0}^{a_{3}}A{\left(\frac{s}{1-ts}\right)}^{k\left(a_{1}c+c+a_{2}+a_{3}\right)+a_{5}}\Gamma \left(k\left(a_{1}c+c+a_{2}+a_{3}\right)+a_{5}\right). \end{array}$$

### 5.5. Characteristic Function

We can obtain the characteristic function for the W-g distribution, from Equation (17), as follows

(22) $$\begin{array}{cc} \phi_{x}\left(t\right)= & E\left(e^{itX}\right)=\sum_{a_{1},a_{2},a_{3},a_{5}=0}^{\infty }\sum_{a_{4}=0}^{a_{3}}A\int_{0}^{\infty }x^{k\left(a_{1}c+c+a_{2}+a_{3}\right)+a_{5}-1)}e^{-x(\frac{1}{s}-it)}dx\\ & =\sum_{a_{1},a_{2},a_{3},a_{5}=0}^{\infty }\sum_{a_{4}=0}^{a_{3}}A{\left(\frac{s}{1-its}\right)}^{k\left(a_{1}c+c+a_{2}+a_{3}\right)+a_{5}}\Gamma \left(k\left(a_{1}c+c+a_{2}+a_{3}\right)+a_{5}\right). \end{array}$$

## 6. Parameter Estimation for Weibull-Gamma Distribution

Assuming a random sample of size *n* is taken from the W-g distribution in Equation (11). Then, to find the maximum likelihood errors (MLEs) of the vector of parameters θ=(c,β,k,s), we need to find the log-likelihood function, then obtain the partial derivative with respect to each parameter and set these derivatives to zero.

The log-likelihood function *ℓ* for the W-g can be given as

(23) $$\begin{array}{cc} \ell = & nlog(c)-nclog(\beta )-nklog(s)-nlog(\Gamma (k))\\ & +\left(k-1\right)\sum_{i=1}^{n}log\left(x_{i}\right)-\frac{1}{s}\sum_{i=1}^{n}x_{i}-\sum_{i=1}^{n}log\left(w\left(x_{i};k\right)\right)\\ & +\left(c-1\right)\sum_{i=1}^{n}log\left(-log\left(w\left(x_{i};k\right)\right)\right)\\ & -\beta^{-c}\sum_{i=1}^{n}{\left(-log(w\left(x_{i};k\right))\right)}^{c} \end{array},$$

where w(x;k) is defined by Equation (14).

The derivatives of Equation (23), with respect to c,β,k, and *s*, respectively, are given by

(24) $$\begin{array}{cc} \frac{\partial \ell }{\partial c}= & \frac{n}{c}-nlog\left(\beta \right)+\sum_{i=1}^{n}log\left(-log\left(w\left(x_{i};k\right)\right)\right)\\ & -\sum_{i=1}^{n}log\left(\frac{-log(w\left(x_{i};k\right))}{\beta }\right){\left(\frac{-log(w\left(x_{i};k\right))}{\beta }\right)}^{c} \end{array},$$

(25) $$\frac{\partial \ell }{\partial \beta }=\frac{-nc}{\beta }+\frac{c}{\beta^{c+1}}\sum_{i=1}^{n}{\left(-log(w(x;k))\right)}^{c},$$

(26) $$\begin{array}{cc} \frac{\partial \ell }{\partial k}= & -nlog\left(s\right)-\frac{n}{\Gamma (k)}\frac{d}{dk}\Gamma \left(k\right)+\sum_{i=1}^{n}log\left(x_{i}\right)-\sum_{i=1}^{n}\frac{\frac{d}{dk}w\left(x_{i};k\right)}{w(x_{i};k)}\\ & +\left(c-1\right)\sum_{i=1}^{n}\frac{\frac{d}{dk}w\left(x_{i};k\right)}{w\left(x_{i};k\right)log\left(w\left(x_{i};k\right)\right)}+\frac{c}{\beta^{c}}\sum_{i=1}^{n}\frac{{\left(-log(w\left(x_{i};k\right))\right)}^{c-1}\frac{d}{dk}w\left(x_{i};k\right)}{w(x_{i};k)},\;\mathrm{and} \end{array}$$

(27) $$\frac{\partial \ell }{\partial s}=\frac{-nk}{s}+\frac{1}{s^{2}}\sum_{i=1}^{n}x_{i}.$$

Thus, the MLEs of the parameters c,β,k, and *s* can be obtained by setting Equations (24)–(27) to zero and solving them iteratively, using numerical methods such as the Newton-Raphson iteration method. Alternatively, the log-likelihood in Equation (23) can be directly maximized, using any standard non-linear optimization tool.

## 7. Simulation Study

This section considers some simulation studies to evaluate the performance of the MLEs of the parameters of the W-g distribution. The simulation is considered over several iterations equal to nsim=1000, and for different sample sizes *n* with the following cases for the true parameters $\theta_{tr}$

- Case I: c=1.5,β=0.5,k=0.5,s=0.4, and
- Case II: c=1.8,β=0.3,k=0.5,s=0.4

The MLE, $\hat{\theta }$, for each parameter can be evaluated using two accuracy measures—the bias and the root mean square error (RMSE)—which can be calculated, respectively, as follows

(28) $$\mathrm{bias}\left(\hat{\theta }\right)=\frac{\sum_{i=1}^{nsim}{\hat{\theta }}_{i}}{nsim}-\theta_{tr}$$

and

(29) $$\mathrm{RMSE}\left(\hat{\theta }\right)=\sqrt{\frac{\sum_{i=1}^{nsim}{\left({\hat{\theta }}_{i}-\theta_{tr}\right)}^{2}}{nsim}}.$$

The Monte Carlo simulation studies were conducted using the R programming language. Table 1 shows the results for the MLE of the parameters of W-g, along with their corresponding average bias and RMSE, respectively. As expected for the method of maximum likelihood, it can be seen that both criteria, bias, and RMSE, generally decreases as the size of the sample *n* increases and the estimates become closer to the true parameters on average.

## 8. Applications

This section illustrates the usefulness of the W-g distribution through five different real data sets. The fit of the W-g is compared with some related distributions; namely the gamma distribution in Equation (2) with shape parameter *k* and scale parameter *s*, and the Weibull distribution in Equation (6) with shape parameter *c* and scale parameter β. The gamma and Weibull distributions are fitted using the “fitdistr” function from the *MASS* package in R. Additionally, the fitting is compared with the EG in Equation (5) with power parameter α, shape parameter *k*, and scale parameter *s*. Also, the data is fitted by the exponentiated exponential (EE), introduced in [22] as an alternative to the gamma and Weibull distributions. The EE is obtained by exponentiating the classical exponential distribution to a power (shape) parameter α as $F\left(x\right)={\left[1-e^{-sx}\right]}^{\alpha }$, where *s* is the scale parameter and α is the shape parameter. The results for EG, EE, and W-g were obtained using the package *Newdistns*, given in [23], in the statistical software R.

In particular, the MLEs of the parameters for each of the distributions with the value of the log-likelihood were computed. Then, to choose the best model among these various models, the Akaike Information Criterion (AIC), given in [24], was computed and the best model is the model with the minimum AIC values. The plots of the expected frequencies for the fitted gamma, Weibull, EE, EG, and W-g were compared with the histograms of the observed frequencies. Furthermore, the empirical cdf was plotted and compared with the estimated cdf for each of the distributions.

### 8.1. First Dataset

First, we will consider the dataset discussed in [25], which concerns with a large system with 30 units, in which the failure and running times are 2.75, 0.13, 1.47, 0.23, 1.81, 0.30, 0.65, 0.10, 3.00, 1.73, 1.06, 3.00, 3.00, 2.12, 3.00, 3.00, 3.00, 0.02, 2.61, 2.93, 0.88, 2.47, 0.28, 1.43, 3.00, 0.23, 3.00, 0.80, 2.45, and 2.66.

**Figure 3:**
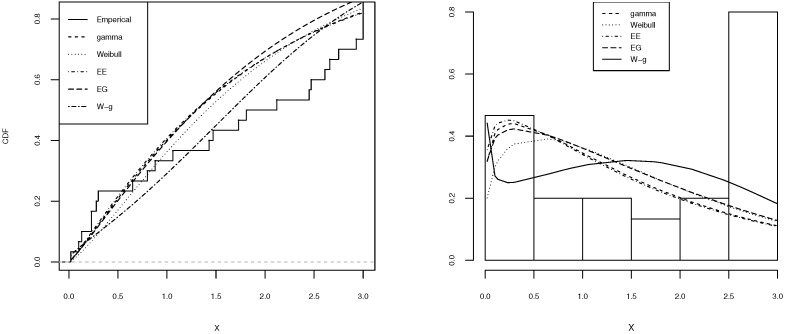
Comparison of W-g distribution with the other distributions for the first dataset. (**Left**): cdf for each of the fitted distributions. (**Right**): observed and expected frequencies for each model.

**Figure 4:**
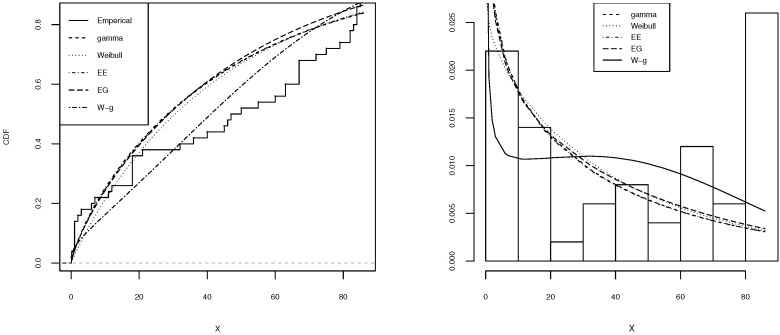
Comparison of W-g distribution with the other distributions for the second dataset. (**Left**): cdf for each of the fitted distributions. (**Right**): observed and expected frequencies for each model.

**Figure 5:**
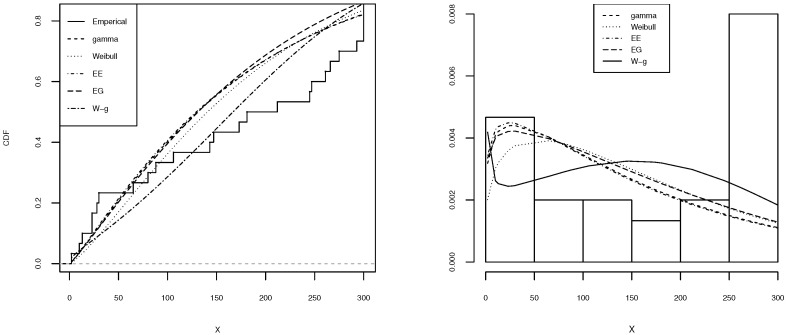
Comparison of W-g distribution with the other distributions for the third dataset. (**Left**): cdf for each of the fitted distributions. (**Right**): observed and expected frequencies for each model.

**Figure 6:**
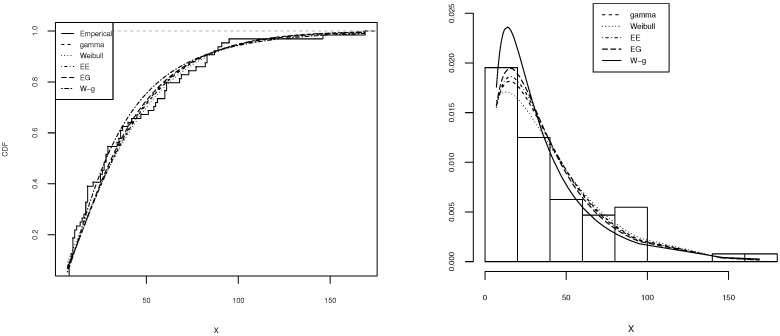
Comparison of W-g distribution with the other distributions for the fourth dataset. (**Left**): cdf for each of the fitted distributions. (**Right**): observed and expected frequencies for each model.

**Figure 7:**
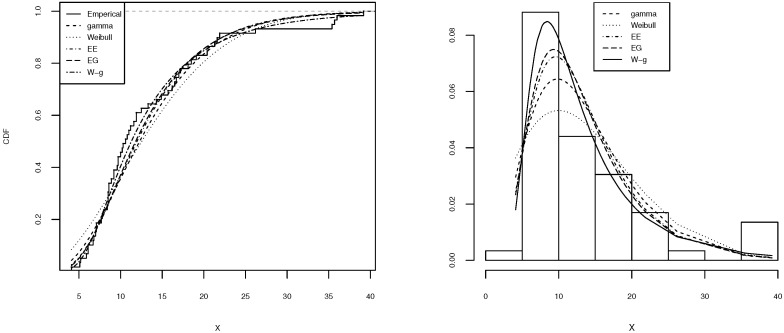
Comparison of W-g distribution with the other distributions for the fifth dataset. (**Left**): cdf for each of the fitted distributions. (**Right**): observed and expected frequencies for each model.

Table 2, Table 3, Table 4, Table 5 and Table 6 shows a summary of the MLEs of the parameters, the log-likelihood, and the AIC for each model. It can be seen that the W-g can be selected as the best model, according to its low AIC when compared to the other fitted distributions. The histogram of the data and plots of the estimated pdf and cdf for each model are displayed in Figure 3, Figure 4, Figure 5, Figure 6 and Figure 7. It is clear that the proposed W-g distribution is the closest to the actual distribution of the data. Therefore, the W-g distribution can be selected as the best model for all datasets.

### 8.2. Second Dataset

The second dataset consists of the lifetimes of n=50 components, given in [26] as: 0.1, 0.2, 1.0, 1.0, 1.0, 1.0, 1.0, 2.0, 3.0, 6.0, 7.0, 11.0, 12.0, 18.0, 18.0, 18.0, 18.0, 18.0, 21.0, 32.0, 36.0, 40.0, 45.0, 46.0, 47.0, 50.0, 55.0, 60.0, 63.0, 63.0, 67.0, 67.0, 67.0, 67.0, 72.0, 75.0, 79.0, 82.0, 82.0, 83.0, 84.0, 84.0, 84.0, 85.0, 85.0, 85.0, 85.0, 85.0, 86.0, and 86.0.

### 8.3. Third Dataset

The third dataset also gives the failure and running times of a sample of n=30 devices, given in [25] as: 2, 10, 13, 23, 23, 28, 30, 65, 80, 88, 106, 143, 147, 173, 181, 212, 245, 247, 261, 266, 275, 293, 300, 300, 300, 300, 300, 300, 300, and 300.

### 8.4. Fourth Dataset

The dataset considered here, discussed by [27], presents the waiting times between 65 consecutive eruptions of a blowhole, called the Kiama Blowhole, as follows: 83, 51, 87, 60, 28, 95, 8, 27, 15, 10, 18, 16, 29, 54, 91, 8, 17, 55, 10, 35,47, 77, 36, 17, 21, 36, 18, 40 , 10, 7, 34, 27, 28, 56, 8, 25, 68, 146, 89, 18, 73, 69, 9, 37, 10, 82, 29, 8, 60, 61, 61, 18, 169, 25, 8, 26, 11, 83, 11, 42, 17, 14, 9, and 12.

### 8.5. Fifth Dataset

The fifth dataset is from [28], and is the monthly actual tax revenues in Egypt between January 2006 and November 2010. These actual taxes, in 1000 million Egyptian pounds, are as follows: 5.9, 20.4, 14.9, 16.2, 17.2, 7.8, 6.1, 9.2, 10.2, 9.6, 13.3, 8.5, 21.6, 18.5, 5.1, 6.7, 17, 8.6, 9.7, 39.2, 35.7, 15.7, 9.7, 10, 4.1, 36, 8.5, 8, 9.2, 26.2, 21.9, 16.7, 21.3, 35.4, 14.3, 8.5, 10.6, 19.1, 20.5, 7.1, 7.7, 18.1, 16.5, 11.9, 7, 8.6, 12.5, 10.3, 11.2, 6.1, 8.4, 11, 11.6, 11.9, 5.2, 6.8, 8.9, 7.1, and 10.8.

## 9. Conclusions

The W-g distribution, a member of the W-G family, is proposed and discussed. This distribution is introduced as a new four-parameter distribution which extends the classical gamma distribution. This generalization can provide more flexibility in analyzing real data. Some special cases of this distribution are presented. Furthermore, some characteristics of this new distribution are obtained. The maximum likelihood method is applied to estimate the model parameters. Different simulation studies are conducted, with different sample sizes, to verify the consistency of the estimates in terms of the bias and RMSE. The results indicate the good performance of the proposed estimators. The usefulness of the suggested distribution is illustrated by means of five real-life datasets. The proposed W-g distribution can consistently provide a better fit than some other common competitive models. Hence, the new W-g distribution can be applied as a competitive model to fit different real data.

## Figures and Tables

**Table 1:** Simulation study: W-g parameter estimates, together with bias and root mean square error (RMSE), for two different cases with different sample sizes. MLE, maximum likelihood error.

Sample Size	Parameter	Case I			Case II		
		MLE	Bias	RMSE	MLE	Bias	RMSE
n=30	*c*	1.8567	0.3567	1.3371	2.4955	0.6955	1.8919
	β	0.9846	0.4846	1.2006	0.8371	0.5371	1.1462
	*k*	0.8473	0.3473	0.9991	0.7038	0.2038	0.7729
	*s*	0.3492	−0.0508	1.0025	0.3349	−0.0651	0.9125
n=100	*c*	1.6269	0.1269	0.8466	1.9732	0.1732	1.0898
	β	0.7970	0.2970	0.8186	0.5303	0.2303	0.6201
	*k*	0.6396	0.1396	0.5129	0.6788	0.1788	0.5824
	*s*	0.4629	0.0629	0.9147	0.3931	−0.0069	0.8175
n=500	*c*	1.5168	0.0168	0.4565	1.7818	−0.0182	0.4919
	β	0.7535	0.2535	0.7530	0.3760	0.0760	0.2767
	*k*	0.5373	0.0373	0.2011	0.5534	0.0534	0.2481
	*s*	0.4577	0.0577	0.4976	0.4053	0.0053	0.4096

**Table 2:** Estimation for the first dataset.

Distribution	gamma	Weibull	EE	EG	W-g
Parameter estimates	$\hat{k}=1.1894$	$\hat{c}=1.265$	$\hat{\alpha }=1.1543$	$\hat{\alpha }=0.0210$	$\hat{c}=6.0638$
	$\hat{s}=1.4884$	$\hat{\beta }=1.8805$	$\hat{s}=1.6231$	$\hat{k}=54.853$	$\hat{\beta }=6.4448$
				$\hat{s}=0.0849$	$\hat{k}=0.0085$
					$\hat{s}=1.891$
Log-likelihood	−46.8656	−46.1587	−46.9569	−44.3009	−42.1281
AIC	97.7311	96.3175	97.9139	94.6018	92.2563

**Table 3:** Estimation for the second dataset.

Distribution	gamma	Weibull	EE	EG	W-g
Parameter estimates	$\hat{k}=0.7991$	$\hat{c}=0.9492$	$\hat{\alpha }=0.7802$	$\hat{\alpha }=0.0655$	$\hat{c}=5.1941$
	$\hat{s}=57.1717$	$\hat{\beta }=44.9466$	$\hat{s}=53.4185$	$\hat{k}=11.8984$	$\hat{\beta }=7.5083$
				$\hat{s}=10.875$	$\hat{k}=0.0044$
					$\hat{s}=39.4314$
Log-likelihood	−240.1902	−241.0018	−239.9952	−237.314	−231.7916
AIC	484.3804	486.0037	483.9903	480.628	471.5832

**Table 4:** Estimation for the third dataset.

Distribution	gamma	Weibull	EE	EG	W-g
Parameter estimates	$\hat{k}=1.1892$	$\hat{c}=1.2651$	$\hat{\alpha }=1.1659$	$\hat{\alpha }=0.0236$	$\hat{c}=6.2924$
	$\hat{s}=148.8595$	$\hat{\beta }=188.0556$	$\hat{s}=161.1306$	$\hat{k}=47.957$	$\hat{\beta }=6.3784$
				$\hat{s}=9.8477$	$\hat{k}=0.0084$
					$\hat{s}=197.8811$
Log-likelihood	−185.0207	−184.3138	−185.113	−182.4996	−180.267
AIC	374.0413	372.6277	374.2259	370.9992	368.5341

**Table 5:** Estimation for the fourth dataset.

Distribution	gamma	Weibull	EE	EG	W-g
Parameter estimates	$\hat{k}=1.6208$	$\hat{c}=1.2744$	$\hat{\alpha }=1.7317$	$\hat{\alpha }=7.3683$	$\hat{c}=0.7086$
	$\hat{s}=24.5738$	$\hat{\beta }=43.205$	$\hat{s}=28.5766$	$\hat{k}=0.2578$	$\hat{\beta }=4.0834$
				$\hat{s}=36.8853$	$\hat{k}=4.6704$
					$\hat{s}=3.6166$
Log-likelihood	−295.8994	−296.9001	−295.666	−295.2987	−293.5914
AIC	595.7988	597.8003	595.332	596.5974	595.1828

**Table 6:** Estimation for the fifth dataset.

Distribution	gamma	Weibull	EE	EG	W-g
Parameter estimates	$\hat{k}=3.6782$	$\hat{c}=1.8404$	$\hat{\alpha }=5.5309$	$\hat{\alpha }=33.1003$	$\hat{c}=0.5883$
	$\hat{s}=3.667$	$\hat{\beta }=15.306$	$\hat{s}=5.5966$	$\hat{k}=0.207$	$\hat{\beta }=2.8474$
				$\hat{s}=7.0303$	$\hat{k}=16.5821$
					$\hat{s}=0.5763$
Log-likelihood	−193.0820	−197.2905	−191.2235	−190.3999	−188.3944
AIC	390.164	398.5811	386.4471	386.7998	384.7887

## Abbreviations

The following abbreviations are used in this manuscript:

cdf	cumulative distribution function
pdf	probability density function
RV	random variable
MLE	maximum likelihood estimator
W-G	Weibull-generated
W-g	Weibull-gamma
EG	exponentiated gamma
EE	exponentiated exponential
AIC	Akaike Information Criterion
RMSE	root mean squared error
